# Diagnostic tracks in emergency departments match discharge diagnoses fairly well

**DOI:** 10.1186/1757-7241-23-S1-A49

**Published:** 2015-07-16

**Authors:** Birgitte Nørgaard, Christian B Mogensen, Lars S Teglbjærg, Mikkel Brabrand, Annmarie T Lassen

**Affiliations:** 1 Institute of Public Health, University of Southern Denmark, Odense, Denmark; 2 Emergency Department, Aabenraa Hospital, Hospital of Southern Jutland, Aabenraa, Denmark; 3 Institute of Regional Health Research, University of Southern Denmark, Odense, Denmark; 4 Emergency Department, OUH Odense University Hospital, Svendborg, Denmark; 5 Emergency Department, Hospital of South West Jutland, Esbjerg, Denmark; 6 Emergency Department, OUH Odense University Hospital, Odense, Denmark

## Background

Patients arrive in the emergency departments with symptoms and problems rather than with diagnoses. Since 2008, the categorisation and subsequent treatment of patients in emergency departments in the Region of Southern Denmark (RSD) have been based on presenting symptoms. Quality and effectiveness is assumed to improve with acceptable diagnostic track assignment.

Our aim was to determine the degree to which assigned diagnostic tracks matched clinically relevant discharge diagnoses.

## Methods

A descriptive cohort study of all patients assigned to a diagnostic track in the five emergency departments between April 1^st ^and June 30^th ^2013 was conducted. Data were linked to data reported to the Danish National Patient Register. Using Delphi method we developed a quality standard to indicate assignment to an acceptable diagnostic track (linkages between diagnostic tracks and discharge diagnosis).

## Results

Of the identified 17,694 patient contacts leading to assignment to a diagnostic track, 16,543 were validated as unique patient pathways. Patients' mean age was 59.8 years; mean length of stay was 3.2 days. The number of different discharge diagnoses used by emergency departments was 1,792; the corresponding number for hospital discharge was 2,030. All 40 diagnostic tracks offered by the region were represented. Sixty-eight per cent had been assigned an acceptable diagnostic track (CI 67.2-68.7). For 30 diagnostic tracks, we found appropriate use of tracks in more than 50% of cases. With stays between 2 and 5 days, significantly more patients were assigned an acceptable diagnostic track compared to shorter or longer stays (p < 0.001). Younger patients were more likely to be offered an acceptable track (p < 0.001). No significant association was established between comorbidity and assignment to an acceptable diagnostic track. Fourteen diagnostic tracks covered 80% of the included pathways.

## Conclusions

We found that 68% of the included patients were assigned an acceptable diagnostic track, and that about 80% of all acute pathways were covered by 14 diagnostic tracks. The studied emergency departments showed no significant differences with respect to their success in assigning patients to acceptable pathways. We suggest further modification and refinement of the concept of diagnostic tracks, followed by testing.

